# Supplementary material for: Development of an AI-Based Predictive Algorithm for Early Diagnosis of High-Risk Dementia Groups among the Elderly: Utilizing Health Lifelog Data
Source: Healthcare (Basel). 2024 Sep 18;12(18):1872. doi: 10.3390/healthcare12181872 (PMC11431183; doi:10.3390/healthcare12181872)
Supplement: Supplementary file 1 [file healthcare-12-01872-s001.zip › healthcare-3180466-supplementary-done.pdf]

healthcare MDPI

1. Logistic Regression Confusion Matrix for Original Data (Gait)

	Predicted: CN	Predicted: MCI+Dem
Actual: CN	26	0
Actual: MCI+Dem	4	3

2. Random Forest Confusion Matrix for Original Data (Gait)

	Predicted: CN	Predicted: MCI+Dem
Actual: CN	22	4
Actual: MCI+Dem	4	3

3. LightGBM Confusion Matrix for Original Data (Gait)

	Predicted: CN	Predicted: MCI+Dem
Actual: CN	26	0
Actual: MCI+Dem	4	3

4. Support Vector Machine Classification Confusion Matrix for Original Data (Gait)

	Predicted: CN	Predicted: MCI+Dem
Actual: CN	26	0
Actual: MCI+Dem	4	3

5. Logistic Regression Confusion Matrix for Original Data (Sleep)

	Predicted: CN	Predicted: MCI+Dem
Actual: CN	26	0
Actual: MCI+Dem	4	3

6. Random Forest Confusion Matrix for Original Data (Sleep)

	Predicted: CN	Predicted: MCI+Dem
Actual: CN	22	4
Actual: MCI+Dem	4	3

7. LightGBM Confusion Matrix for Original Data (Sleep)

	Predicted: CN	Predicted: MCI+Dem
Actual: CN	25	1
Actual: MCI+Dem	4	3

8. Support Vector Machine Classification Confusion Matrix for Original Data (Sleep)

	Predicted: CN	Predicted: MCI+Dem
Actual: CN	26	0
Actual: MCI+Dem	4	3

9. Logistic Regression Confusion Matrix for Augmented Data (Gait)

	Predicted: CN	Predicted: MCI+Dem
Actual: CN	21	5
Actual: MCI+Dem	4	3

10. Random Forest Confusion Matrix for Augmented Data (Gait)

	Predicted: CN	Predicted: MCI+Dem
Actual: CN	22	4
Actual: MCI+Dem	3	4

11. LightGBM Confusion Matrix for Augmented Data (Gait)

	Predicted: CN	Predicted: MCI+Dem
Actual: CN	24	2
Actual: MCI+Dem	4	3

12. Support Vector Machine Classification Confusion Matrix for Augmented Data (Gait)

	Predicted: CN	Predicted: MCI+Dem
Actual: CN	26	0
Actual: MCI+Dem	4	3

13. Logistic Regression Confusion Matrix for Augmented Data (Sleep)

	Predicted: CN	Predicted: MCI+Dem
Actual: CN	23	3
Actual: MCI+Dem	3	4

14. Random Forest Confusion Matrix for Augmented Data (Sleep)

	Predicted: CN	Predicted: MCI+Dem
Actual: CN	22	4
Actual: MCI+Dem	3	4

15. LightGBM Confusion Matrix for Augmented Data (Sleep)

	Predicted: CN	Predicted: MCI+Dem
Actual: CN	26	0
Actual: MCI+Dem	7	0

16. Support Vector Machine Classification Confusion Matrix for Augmented Data (Sleep)

	Predicted: CN	Predicted: MCI+Dem
Actual: CN	26	0
Actual: MCI+Dem	4	3
